# High prevalence of posttraumatic stress disorder in survivors of aneurysmal subarachnoid hemorrhage: a 6-month follow-up screening study

**DOI:** 10.1007/s13760-026-02989-y

**Published:** 2026-03-07

**Authors:** Isabela Costola Windlin, Bruno Braga Sisnando da Costa, Pedro Henrique Mouty Rabello, Ilan David Friedmann, Yan Luca de Abreu Alves Jacinto, Davi Jorge Fontoura Solla, Carlos Gilberto Carlotti Jr., Eberval Gadelha Figueiredo

**Affiliations:** 1 Department of Neurosurgery, University of São Paulo, São Paulo, Brazil; 2 Division of Neurosurgery, School of Medicine, University of São Paulo (FMUSP), Hospital das Clínicas/FMUSP - Rua Dr. Enéas de Carvalho Aguiar, 255, São Paulo, 05403-010 - 28 SP Brazil

**Keywords:** Subarachnoid hemorrhage, Posttraumatic stress disorder, Neuropsychiatric outcomes, Anxiety, Depression

## Abstract

**Background:**

Posttraumatic stress disorder (PTSD) is increasingly recognized as a sequela of subarachnoid hemorrhage (SAH); however, its prevalence and clinical correlates remain understudied. This study assessed the prevalence of PTSD symptoms and associated psychological symptoms among survivors of SAH.

**Methods:**

A 6-month prospective analysis of 45 SAH survivors from the GASH trial (NCT03569540) was conducted. PTSD symptoms were evaluated using the Screen for Posttraumatic Stress Symptoms (SPTSS), with concurrent assessment of anxiety/depression (Hospital Anxiety and Depression Scale, HADS) and functional outcomes (modified Rankin Scale).

**Results:**

At six months post-SAH, 55,56% (*n* = 25) of patients met the SPTSS criteria for significant PTSD symptoms. Avoidance (mean score: 23,36 ± 11,75) and hyperarousal (19,52 ± 10,22) were the most prominent symptoms. PTSD was strongly associated with concurrent anxiety (HADS-A ≥ 7; *p* < 0.001) and depression (HADS-D ≥ 6; *p* < 0.001).

**Conclusion:**

Nearly half of SAH survivors screened positive for PTSD-related symptoms within six months, with avoidance behaviors predominating. The high comorbidity of anxiety and depression underscores the necessity for integrated neuropsychiatric care in SAH follow-up protocols.

## Introduction

Posttraumatic stress disorder (PTSD) is a debilitating psychiatric condition that may occur following exposure to a traumatic event involving actual or threatened death, serious injury, or sexual violence. It is defined by clusters of symptoms including intrusive recollections, avoidance, negative alterations in cognition and mood, and hyperarousal, persisting for more than one month after the trauma, according to DSM-5-TR criteria [1]. While PTSD has been historically associated with combat, assault, or disasters, recent research underscores that medical events—such as myocardial infarction, stroke, or subarachnoid hemorrhage—can also serve as psychologically traumatic experiences capable of precipitating PTSD [2, 3].

Subarachnoid hemorrhage (SAH) is a neurologic emergency characterized by the extravasation of blood into the subarachnoid space, most commonly due to rupture of an intracranial aneurysm, but can occur as a result of vascular malformations or vasculitis [4]. Although it accounts for only 5% of all strokes, SAH disproportionately affects younger patients and carries a high burden of morbidity and mortality, with an incidence of approximately nine cases per 100,000 person-years and a fatality rate of around 40%, despite modern treatment [4–6]. The acute clinical presentation—often involving sudden-onset severe headache, loss of consciousness, and neurocritical care interventions—can be highly traumatic, and many survivors report persistent neuropsychiatric sequelae, including anxiety, depression, and cognitive deficits [4, 7, 8].

Increasing attention has been directed toward the psychological aftermath of SAH, including its potential to act as a precipitating event for posttraumatic stress disorder (PTSD), particularly in those exposed to perceived life threat, passive coping style, prolonged intensive care and previous psychiatric disorders [3, 9].

From a neurobiological standpoint, the core of the fear circuitry implicated in posttraumatic stress disorder lies within the amygdala–hippocampal–prefrontal network. The amygdala plays a central role in detecting threat and generating the autonomic and behavioral components of fear, while simultaneously enhancing the emotional salience of trauma-related memories encoded in cortical regions. Through rapid subcortical pathways that bypass the neocortex, the amygdala can trigger a “fight-or-flight” response before conscious appraisal, promoting immediate survival-oriented reactions. However, persistent amygdala hyperreactivity, coupled with deficient top-down inhibition from the prefrontal cortex, contributes to sustained hyperarousal and exaggerated startle responses. In parallel, abnormal hippocampal modulation of contextual memory processing fosters overgeneralization of threat cues, leading individuals to avoid reminders of the trauma to prevent reactivation of distressing emotions. These mechanisms underpin the characteristic patterns of avoidance and hyperarousal that define the clinical expression of PTSD and perpetuate chronic stress dysregulation [10].

Despite growing recognition of the psychological impact of subarachnoid hemorrhage, PTSD remains underdiagnosed and undertreated in this population. Affected individuals may present with subtle or atypical symptoms that overlap with post-stroke fatigue, cognitive impairment, or depression, complicating clinical detection [11]. Furthermore, PTSD following SAH has been associated with poorer functional outcomes, reduced quality of life, and diminished adherence to secondary prevention measures [3, 12]. Identifying patients at risk and understanding the true prevalence of PTSD symptoms in SAH survivors is therefore essential for developing targeted interventions and improving long-term recovery.

While prior studies have explored psychiatric morbidity after SAH, data on PTSD prevalence remain limited and heterogeneous, often constrained by variable diagnostic criteria and inconsistent follow-up durations [3]. In this context, further research is needed to clarify the burden of PTSD in this group and inform neuropsychological screening protocols in the post-acute phase. The present study aims to assess the prevalence of significant PTSD symptoms in patients who have experienced subarachnoid hemorrhage.

## Methods

This study involved patients enrolled in the clinical trial ‘GASH’ (Glibenclamide in Aneurysmal Subarachnoid Hemorrhage), which was previously published and registered on ClinicalTrials.gov (NCT03569540) on June 19, 2018 [13]. Initially, 78 patients aged 18 to 70 years, diagnosed with SAH through radiological confirmation and presenting within 96 h of symptom onset, were included in the trial.

In compliance with institutional ethical guidelines, all participants underwent definitive treatment for their intracranial aneurysm (either microsurgical or endovascular) and received nimodipine (60 mg) every four hours for 21 days.

According to the GASH protocol [13], patients were randomly assigned in a 1:1 ratio to receive either 5 mg of glibenclamide or a placebo (starch). Treatment began after aneurysm repair and within 96 h of the bleeding event. Each patient received the assigned intervention for 21 days, administered as one 5 mg tablet daily orally or via a nasogastric tube.

For this study, patients who completed the medication protocol were evaluated in person by a psychologist blinded to treatment allocation and clinical course at the Neurology Outpatient Clinic of Hospital das Clínicas, University of São Paulo, six months after hospital discharge, to assess cognitive function, mood, and quality of life. Upon inclusion in the trial, all available contact information (telephone numbers, email addresses, and home addresses) was collected and recorded. Patients were later contacted by telephone, email, or mail and invited for the follow-up evaluation. When possible, the assessment coincided with their scheduled neurosurgical outpatient appointment. For those already discharged, a specific appointment was arranged; for patients who remained hospitalized, the evaluation was conducted at the bedside.

The evaluation tools included the Hospital Anxiety and Depression Scale (HADS) and the Screen for Posttraumatic Stress Symptoms (SPTSS), along with a functional assessment using the modified Rankin Scale. The anxiety and depression questionnaires were interviewer-administered, and no questionnaire items were missing. Since the SPTSS is a screening tool, it identifies individuals with significant PTSD-related symptoms rather than those with a confirmed diagnosis of the disorder. All patients who scored above the cutoff points on the psychological symptom scales were appropriately counseled and referred to public mental health services (psychosocial support centers or primary care units), following the referral and counter-referral system of the Brazilian Unified Health System (SUS).

### Inclusion criteria

The inclusion criteria for this study were: radiologically confirmed diagnosis of subarachnoid hemorrhage (SAH); age between 18 and 70 years; and clinical presentation within 96 h of ictal onset.

### Exclusion criteria

The exclusion criteria were: prior use of glibenclamide during the ictal event; pregnancy; known pre-existing renal or hepatic disease; patients who were not fully independent before the hemorrhage; patients with no expected survival due to the severity of their clinical condition; presence of severe comorbidities likely to affect survival; strong suspicion of drug use or alcohol abuse; prior use of anticoagulant medication; and patients with neurocognitive or functional impairment that could hinder the proper execution of the study assessments.

### Statistical analysis

The sample size was predefined as 80 patients, deemed adequate to estimate the prevalence of posttraumatic stress disorder (PTSD) symptoms in patients after subarachnoid hemorrhage, with a 95% confidence interval and an estimated precision of approximately 10%.

Statistical analyses were performed using JASP software, version 0.19.3.0 (JASP Team, University of Amsterdam, The Netherlands). Associations between the presence of posttraumatic stress disorder (PTSD)-related symptoms and psychological distress (anxiety and depression), as well as comparisons between PTSD-positive and PTSD-negative groups with respect to sex, were assessed using contingency analyses with the chi-square test. Differences in years of schooling between groups were analyzed using the Fisher-Freeman-Halton exact test.

For quantitative variables, either the Student’s *t*-test or the Mann–Whitney *U* test was applied, depending on data distribution normality, as assessed by the Shapiro–Wilk test. The Student’s *t*-test was used for age, while the Mann–Whitney *U* test was applied to the modified Rankin Scale (mRS) scores at hospital discharge and at six months. A 95% confidence interval was adopted, and the Wilson method was used to calculate the confidence intervals for the prevalences reported in the study.

No adjustments for multiple comparisons were applied, as the analyses were exploratory in nature.

## Results

**Fig. 1:**
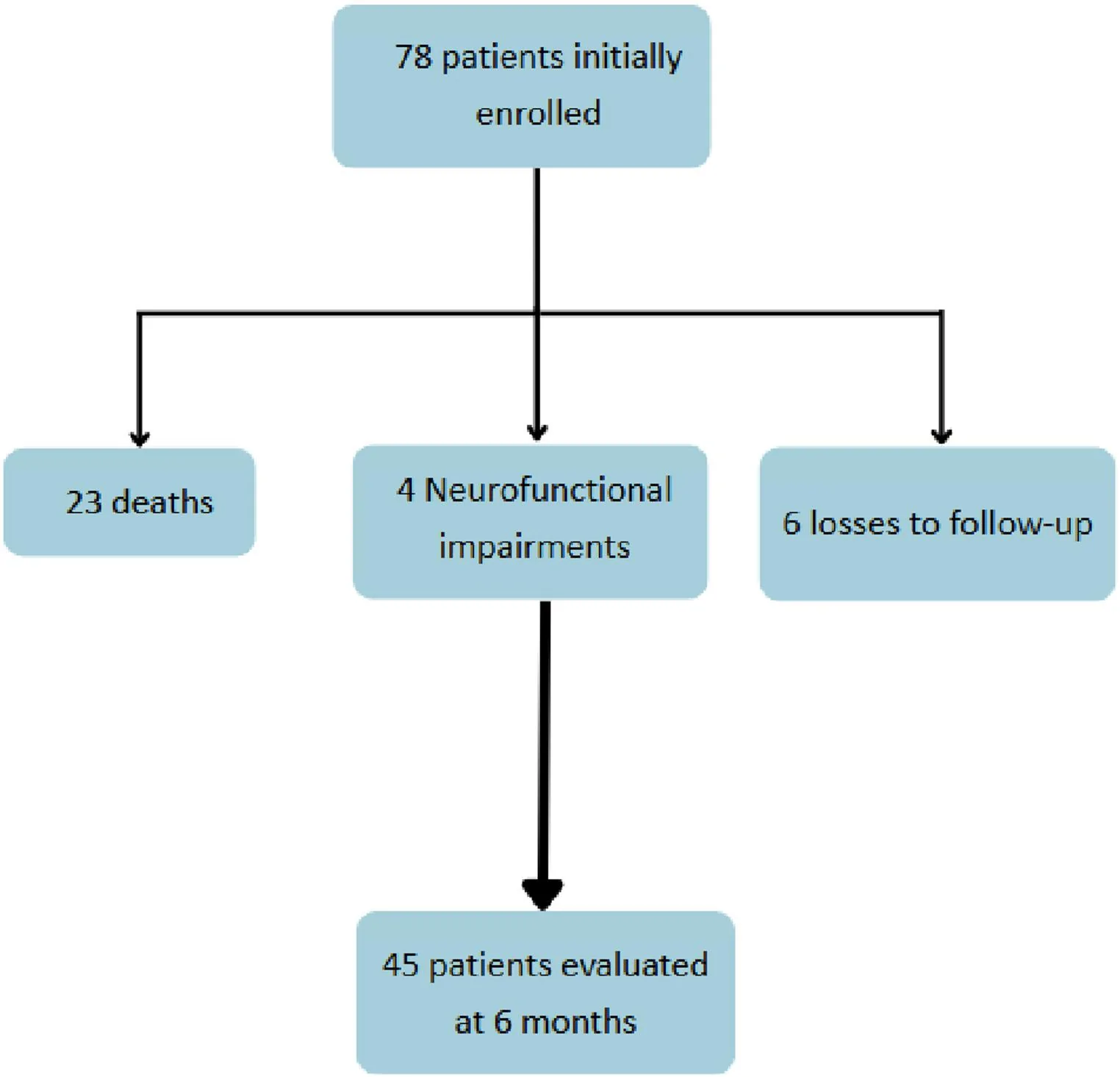
Participants flow from initial enrollment through 6-month assessment, including attrition due to death, neurofunctional disability, and loss to follow-up

Of the 78 patients initially included in the study, 45 completed the 6-month follow-up assessment and were assessed for the presence of Posttraumatic Stress Disorder (PTSD)-related symptoms. The patient distribution is presented in Table 1; Fig. 1.

**Table 1:** Distribution of deaths, exclusions, and losses to follow-up from the initial cohort (*n* = 78)

	Overall (78)
Deaths	23 (29,5%)
Exclusions due to neurofunctional impairments (Expressive aphasia, global aphasia, motor aphasia with hemiparesis and severe comprehensive difficulty)	4 (5,1%)
Losses to follow-up	6 (7,7%)

**Note:** Values are presented as n (%)

Six months after the occurrence of aneurysmal subarachnoid hemorrhage, 55,6% (*n* = 25; 95% CI 40,1–70,1%) of the patients in the total sample presented with PTSD-related symptoms.

Among the 45 participants assessed using the Screening Tool for Posttraumatic Stress Symptoms (SPTSS), 25 individuals (55,6%) screened positive for significant PTSD-related symptoms (SPTSS ≥ 5). Analysis of symptom domains revealed that avoidance was the most prominent symptom cluster, with a mean score of 23,36 (± 11,75), followed by hyperarousal with a mean score of 19,52 (± 10,22), and re-experiencing with a mean score of 11,16 (± 11,49) (Table 2). These findings suggest a notable burden of PTSD-related symptoms in the study population, particularly characterized by avoidance and hyperarousal.

**Table 2:** Prevalence and distribution of post-traumatic stress symptoms assessed by the SPTSS (*n* = 45)

Variable	Overall (45)
SPTSS ≥ 5	25 (55,56%; 95% CI 40,1–70,1%)
Predominant Symptoms (mean ± SD)	
Avoidance	23,36 ± 11,75
Hyperarousal	19,52 ± 10,22
Re-experiencing	11,16 ± 11,49

Note: SPTSS: Screening Tool for Post-Traumatic Stress Symptoms; CI: confidence interval; SD: standard deviation

The modified Rankin Scale (mRS) scores at 6 months were higher among patients who screened positive for PTSD-related symptoms compared with those who did not (**Mann–Whitney U test**, *p* = 0.008) (Table 3). This finding suggests that, in our sample, patients with Posttraumatic Stress Disorder - related symptoms exhibited greater functional impairment.

**Table 3:** Demographic and clinical characteristics of patients with positive versus negative screening for PTSD-related symptoms at 6 months

Variables	PTSD screening–positive (25)	PTSD screening–negative (20)	*p* value	95% CI
Age	50,88 ± 9,97	48,7 ± 14,1	0,828	2,18 (− 5,13 a 9,49)
Sex	84% Female	60% Female	0,070	3,50 (0,87–14,11)
Education level	0 to < 4: 11 4 to < 8: 6 8 to < 12: 8 > 12: 0	0 to < 4: 9 4 to < 8: 3 8 to < 12: 5 > 12: 3	0,240	
mRS at the time of hospital discharge (mean ± SD)	2,64 ± 1,73	2,05 ± 1,23	0,281	0,59 (− 0,28 a 1,46)
mRS after 6 months (median with interquartile range)	1 (0–2)	0 (0–0,5)	0,008	
Positive for anxiety (HADS-A ≥ 7)	68% (95% CI 48–83%)	10% (95% CI 3–30%)	< 0,001	19.1 (3.6–101.5)
Positive for depression (HADS-D ≥ 6)	76% (95% CI 56–89%)	15% (95% CI 5–36%)	< 0,001	17.9 (3.9–82.1)

**Note:** Data are presented as mean ± standard deviation (SD) for normally distributed continuous variables and as median [interquartile range, IQR] for non-normally distributed variables. Continuous variables were compared between groups using Student’s *t* test or the Mann–Whitney *U* test, as appropriate. Categorical variables are presented as percentages and were compared using the chi-square test or the Fisher–Freeman–Halton exact test, as appropriate (education level). Effect estimates are reported as mean differences for continuous variables and as odds ratios (ORs) for binary categorical variables, with corresponding 95% confidence intervals (95% CI), where applicable

**Abbreviations:** PTSD, posttraumatic stress disorder; mRS, modified Rankin Scale; HADS-A, Hospital Anxiety and Depression Scale–Anxiety; HADS-D, Hospital Anxiety and Depression Scale–Depression; SD, standard deviation; IQR, interquartile range

Furthermore, there was a significant association (**χ2 test**, p < 0.001; Table 3) between screening positive for PTSD-related symptoms and psychological distress. On the HADS, both anxiety (HADS-A ≥ 7) and depression (HADS-D ≥ 6) were significantly associated with a positive PTSD screening.”

## Discussion

Six months after experiencing an aneurysmal subarachnoid hemorrhage, more than half (55,56%) of the patients in our study who completed follow-up showed PTSD-related symptoms, as measured by the Screen for Posttraumatic Stress Symptoms (SPTSS). Among the different PTSD symptom domains, avoidance behaviors were the most prominent, followed by hyperarousal and, less frequently, intrusive symptoms. PTSD-related symptoms were strongly associated with concurrent anxiety and depression.

When considering only the subset of SAH survivors who completed the 6-month follow-up, the prevalence of PTSD-related symptoms we observed was higher than the rates typically reported in the broader stroke population. A systematic review of 30 studies involving 4,320 stroke survivors described a median PTSD prevalence of 17.5%, with the highest rates following SAH (37.1%) and the lowest after transient ischemic attack (TIA, 4.6%) [14]. Another meta-analysis of 1,138 stroke and TIA survivors reported a pooled prevalence of 23% within the first post-event year, decreasing to 11% thereafter. The comparatively higher proportion identified in our followed cohort may reflect the particularly traumatic nature of aneurysmal SAH and is consistent with prior findings showing persistent PTSD in up to 37% of SAH survivors one year after the event [15].

Neurobiological and psychological factors also appear to influence PTSD risk. A large prospective study identified stroke severity, white matter lesion volume, and hippocampal connectivity as correlates of PTSD severity, while infarct volume and location had no effect. High-anxious and defensive coping styles were linked to a more than sixfold increased risk of PTSD, independent of other factors [16]. Similarly, in SAH populations, maladaptive coping has consistently emerged as the strongest predictor of PTSD, rather than demographic or clinical factors [17]. In our cohort, patients who screened positive for PTSD-related symptoms also had higher mRS scores at 6 months, indicating a significant association between PTSD symptom burden and lower functional status, even when accounting for clinical severity and other medical predictors.

The psychological burden of PTSD after SAH extends beyond core symptoms. Prior research has shown that PTSD in this context is strongly associated with fatigue, poor sleep quality, and fear of recurrence—factors that further impair recovery and reduce quality of life. Our findings reinforce the growing consensus that PTSD is a critical factor associated with the long-term well-being of SAH survivors, even in the absence of major neurological deficits [18, 19].

Our findings suggest that integrating systematic PTSD screening and psychological support into routine post-SAH care could improve long-term recovery. Early identification of emotional distress, combined with tailored interventions, may help mitigate fatigue, sleep disturbances, and fear of recurrence, ultimately enhancing patients’ quality of life and functional outcomes.

Implementing tailored interventions requires a multifaceted approach that addresses the specific neurocognitive and emotional needs of SAH survivors. First, routine screening using validated tools (such as the SPTSS or PCL-5) should be integrated into standard neurosurgical follow-up to ensure early detection. For patients who screen positive, Trauma-Focused Cognitive Behavioral Therapy (TF-CBT) is currently considered the gold standard for reducing PTSD symptom severity [20]. Additionally, Eye Movement Desensitization and Reprocessing (EMDR) has emerged as a promising alternative for survivors of acquired brain injury; EMDR may be particularly advantageous for SAH patients with residual linguistic or cognitive deficits (e.g., aphasia), as it places fewer demands on verbal articulation and working memory than traditional talk therapies [21, 22]. Finally, psychoeducation provided to patients and caregivers prior to discharge can help normalize emotional sequelae, reducing the stigma of ‘fear of recurrence’ and encouraging earlier help-seeking behaviors.

Taken together, these results underscore the need for routine PTSD screening and early psychological intervention following SAH, particularly in patients displaying maladaptive coping strategies or signs of emotional distress. Addressing these issues proactively may significantly improve emotional well-being and overall quality of life in this vulnerable population.

### Limitations

Several limitations should be considered when interpreting these findings. First, the attrition rate may have introduced selection bias, as patients with severe disability or cognitive impairment were disproportionately excluded, potentially leading to an underestimation of the true prevalence of PTSD (Table 1). In addition, no formal comparison of baseline demographic or clinical characteristics was performed between patients who completed follow-up and those who were lost, which limits the ability to assess the presence and direction of attrition-related bias.

Second, although the SPTSS is a validated screening instrument, it does not establish a formal PTSD diagnosis. Future studies should incorporate clinician-administered assessments (e.g., CAPS-5) to enhance diagnostic accuracy.

Third, the single-center design and modest sample size limit generalizability. Crucially, the cross-sectional nature of the analysis limits our ability to infer causality between PTSD symptoms and functional outcomes (mRS). Potential confounding factors, such as initial illness severity (e.g., Hunt-Hess or Fisher grades) and pre-existing psychiatric history, warrant consideration. Although we excluded patients with severe neurofunctional impairment, it is plausible that higher initial disease severity contributes independently to both the traumatic experience and persistent functional limitations. Similarly, pre-existing anxiety or depression—which were not systematically assessed at baseline—could predispose patients to developing PTSD symptoms while simultaneously affecting their perception of recovery and quality of life. Future longitudinal studies controlling for these baseline variables are needed to disentangle these associations.

Fourth, the achieved sample size (*n* = 45) was smaller than the planned target of 80 participants. Consequently, the precision assumptions underlying the original study design were not met. This reduction in sample size decreased statistical power, as reflected in the wide confidence interval for the estimated prevalence. Therefore, the results should be interpreted with appropriate caution.

Finally, the 6-month follow-up limits the ability to assess long-term trajectories of PTSD symptoms. Future longitudinal studies are needed to determine whether symptoms persist, fluctuate, or remit over time. Despite these limitations, our findings provide preliminary yet compelling evidence of the substantial burden of PTSD-related symptoms in SAH survivors.

### Risk of bias

While the Limitations section describes the main methodological constraints of the study, this section specifically addresses the *direction and potential impact* of bias introduced by loss to follow-up.

The loss of approximately 42% of the initial cohort may have biased the estimated prevalence of PTSD-related symptoms toward underestimation. As shown in Tables 1, 27 of the 33 patients lost to follow-up either died or had significant neurofunctional impairment, a subgroup plausibly at higher risk for psychological morbidity. Consequently, the observed prevalence likely reflects a healthier survivor population and should not be extrapolated to all patients with aneurysmal subarachnoid hemorrhage.

## Conclusion

This study demonstrated a strikingly high prevalence of PTSD-related symptoms (55,56%) among SAH survivors that completed the 6-month follow-up, with avoidance behaviors being the most prominent symptom. The strong association between PTSD-related symptoms and concurrent anxiety/depression highlights the need for integrated neuropsychiatric care. These findings emphasize the critical need for systematic PTSD screening and integrated mental health care in SAH follow-up protocols, addressing symptoms that are closely linked to long-term patient recovery.

## Data Availability

The datasets generated and analyzed during the current study are available from the corresponding author upon reasonable request.

## References

[1] American Psychiatric Association (2022) Diagnostic and statistical manual of mental disorders. 5th ed, text revision. Washington, DC: APA Publishing

[2] Tedstone JE, Tarrier N (2003) Posttraumatic stress disorder following medical illness and treatment. Clin Psychol Rev 23(3):409–48. 10.1016/s0272-7358(03)00031-x 12729679

[3] Tang WK, Wang L, Tsoi, Kelvin K F.1,2;, Kim JS 3. Post-Traumatic Stress Disorder after Subarachnoid Hemorrhage: A Systematic Review. Neurology India 71(1):p 9–19, Jan–Feb 2023 10.4103/0028-3886.370451 36861567

[4] Lawton MT, Vates GE (2017) Subarachnoid hemorrhage. N Engl J Med 377(3):257–266. 10.1056/NEJMcp1605827 28723321

[5] Mahlamäki K, Rautalin I, Korja M (2022) Case fatality rates of subarachnoid hemorrhage are decreasing with substantial between-country variation: a systematic review of population-based studies between 1980 and 2020. Neuroepidemiology 56(6):402–412. 10.1159/000526983 36282049

[6] RINKER JR et al (2011) Long-term outcomes of patients with aneurysmal subarachnoid haemorrhage: a population-based study. The Lancet Neurology,, pp 349–356. [S.l.], v. 10n. 4 10.1016/S1474-4422(11)70017-5 21435599

[7] Connolly ES, Rabinstein AA, Carhuapoma JR et al (2012) <article-title update="added">Guidelines for the management of aneurysmal subarachnoid hemorrhage: a guideline for healthcare professionals from the American Heart Association/American Stroke Association. Stroke 43(6):1711–1737 10.1161/STR.0b013e3182587839 22556195

[8] Morris PG, Wilson PD, Lindsay Ph JT, Dunn D (2004) Anxiety and depression after spontaneous subarachnoid hemorrhage. Neurosurgery 54(1):47–54 10.1227/01.neu.0000097198.94828.e1 14683540

[9] Visser-Meily JMA, Rinkel GJE, Vergouwen MDI, Passier PECA, van Zandvoort MJE, Post MWM (2013) Post-Traumatic stress disorder in patients 3 years after aneurysmal subarachnoid haemorrhage. Cerebrovasc Dis 1 Oct 36(2):126–130 10.1159/000353642 24029667

[10] Vieweg VR, Julius DA, Fernandez A, Beatty-Brooks M, Hettema JM, Pandurangi AK (2006) Posttraumatic stress disorder: clinical features, pathophysiology, and treatment. Am J Med 119(5):383–390. 10.1016/j.amjmed.2005.09.027 16651048

[11] KIRBY NWAFORDC, Brandon D, RALSTON, Jacob D, COLANTONIO, Mark A, IBEKWE E (2023) LUCKE-WOLD, Brandon. Neurocognitive sequelae and rehabilitation after subarachnoid hemorrhage: optimizing outcomes. J Vascular Dis Basel V 2(2):197–211

[12] Kronish IM, Edmondson D, Li Y, Cohen BE (2012) Post-traumatic stress disorder and medication adherence: results from the mind your heart study. J Psychiatr Res 46(12):1595–1600 10.1016/j.jpsychires.2012.06.011 PMC3485414 22809686

[13] da Costa BBS, Windlin IC, Koterba E, Yamaki VN, Rabelo NN, Solla DJF, Teixeira MJ, Figueiredo EG (2019) Glibenclamide in aneurysmatic subarachnoid hemorrhage (GASH): study protocol for a randomized controlled trial. Trials 20(1):413 10.1186/s13063-019-3517-y PMC6617901 31288831

[14] Janssen EPJ, Spauwen PJJ, Bus BAA, Rijnen SJM, Ponds RWHM (2024) Prevalence of posttraumatic stress disorder after stroke: a systematic literature review. J Psychosom Res 187:111914. 10.1016/j.jpsychores.2024.111914 39306898

[15] Edmondson D, Richardson S, Fausett JK, Falzon L, Howard VJ, Kronish IM (2013) Prevalence of PTSD in survivors of stroke and transient ischemic attack: a meta-analytic review. PLoS One 8(6):e66435. 10.1371/journal.pone.0066435 PMC3686746 23840467

[16] Ben Assayag E, Tene O, Korczyn AD, Solomon Z, Bornstein NM, Shenhar-Tsarfaty S, Seyman E, Niry D, Molad J, Hallevi H (2022) Posttraumatic stress symptoms after stroke: the effects of anatomy and coping style. Stroke 53(6):1924–1933 Epub 2022 Mar 10. PMID: 35264011 10.1161/STROKEAHA.121.036635 35264011

[17] Visser-Meily JM, Rinkel GJ, Vergouwen MD, Passier PE, van Zandvoort MJ, Post MW (2013) Post-traumatic stress disorder in patients 3 years after aneurysmal subarachnoid haemorrhage. Cerebrovasc Dis 36(2):126–30 10.1159/000353642 24029667

[18] Noble AJ, Baisch S, Covey J, Mukerji N, Nath F, Schenk T (2011) Subarachnoid hemorrhage patients’ fears of recurrence are related to the presence of posttraumatic stress disorder. Neurosurgery 69(2):323–32 10.1227/NEU.0b013e318216047e 21415779

[19] Noble AJ, Baisch S, Mendelow AD, Allen L, Kane P, Schenk T (2008) Posttraumatic stress disorder explains reduced quality of life in subarachnoid hemorrhage patients in both the short and long term. Neurosurgery 63(6):1095–104. 10.1227/01.NEU.0000327580.91345.78 19057321

[20] American Psychological Association (2017) Clinical Practice Guideline for the Treatment of Posttraumatic Stress Disorder (PTSD) in Adults. American Psychological Association, Washington, DC

[21] Guina J, Guina C (2018) Wants talk psychotherapy but cannot talk: EMDR for Post-stroke depression with expressive aphasia. Innov Clin Neurosci 15(1–2):45–48 PMID: 29497580; PMCID: PMC5819721 PMC5819721 29497580

[22] Smart CM (2022) Eye movement desensitization and reprocessing for post-stroke post-traumatic stress disorder: case report using the three-phase approach. Brain Inj 36:1372–1381. 10.1080/02699052.2022.2140833 36372972

